# Supplementary material for: ERRATUM: Hypertensive patients with and without kidney disease: assessment of risk factors
Source: Rev Esc Enferm USP. 2024 Oct 4;58:e2024ER14. doi: 10.1590/1980-220X-REEUSP-2024-ER14en (PMC11460653; doi:10.1590/1980-220X-REEUSP-2024-ER14en)
Supplement: Translation

# Translation: ERRATA: Hipertensos com e sem doença renal: avaliação de fatores de risco

**DOI:** 10.1590/1980-220X-REEUSP-2024-ER14pt

No artigo “Hipertensos com e sem doença renal: avaliação de fatores de risco”, com número de: DOI: https://doi.org/10.1590/S0080-623420150000700015, publicado na Revista da Escola de Enfermagem da USP, volume 49 (spe) de 2015, p. 101–108, na página 108:

**Leia-se:**

## Apoio financeiro

O presente trabalho foi realizado com apoio da Coordenação e Aperfeiçoamento de Pessoal de Nível Superior – Brasil (CAPES) – Código de Financiamento 001.
